# CluGene: A Bioinformatics Framework for the Identification of Co-Localized, Co-Expressed and Co-Regulated Genes Aimed at the Investigation of Transcriptional Regulatory Networks from High-Throughput Expression Data

**DOI:** 10.1371/journal.pone.0066196

**Published:** 2013-06-18

**Authors:** Tania Dottorini, Pietro Palladino, Nicola Senin, Tania Persampieri, Roberta Spaccapelo, Andrea Crisanti

**Affiliations:** 1 Department of Biological Sciences, Imperial College London, London, United Kingdom; 2 Department of Experimental Medicine, University of Perugia, Perugia, Italy; 3 Department of Industrial Engineering, University of Perugia, Perugia, Italy; Kyushu Institute of Technology, Japan

## Abstract

The full understanding of the mechanisms underlying transcriptional regulatory networks requires unravelling of complex causal relationships. Genome high-throughput technologies produce a huge amount of information pertaining gene expression and regulation; however, the complexity of the available data is often overwhelming and tools are needed to extract and organize the relevant information. This work starts from the assumption that the observation of *co-occurrent* events (in particular co-localization, co-expression and co-regulation) may provide a powerful starting point to begin unravelling transcriptional regulatory networks. Co-expressed genes often imply shared functional pathways; co-expressed and functionally related genes are often co-localized, too; moreover, co-expressed and co-localized genes are also potential targets for co-regulation; finally, co-regulation seems more frequent for genes mapped to proximal chromosome regions. Despite the recognized importance of analysing co-occurrent events, no bioinformatics solution allowing the simultaneous analysis of co-expression, co-localization and co-regulation is currently available. Our work resulted in developing and valuating CluGene, a software providing tools to analyze multiple types of co-occurrences within a single interactive environment allowing the interactive investigation of combined co-expression, co-localization and co-regulation of genes. The use of CluGene will enhance the power of testing hypothesis and experimental approaches aimed at unravelling transcriptional regulatory networks. The software is freely available at http://bioinfolab.unipg.it/.

## Introduction

The maintenance and modulation of cellular activities is a complex process controlled by the co-ordinated expression and action of different gene networks. This composite process is regulated by a higher-order genome structure arranged into transcriptional regulatory modules. Understanding the organization of these networks is crucial for elucidating how different gene combinations work together in accomplishing distinct cellular functions. Although genome-wide transcriptional analysis and in general genome high-throughput experimental technologies are producing a vast amount of information, the relationships existing amongst genome organization, gene regulation and the diverse facets of cell activity still remain largely unknown.

Experimental observations indicate that genes sharing similar expression patterns are correlated either from a functional or evolutionary standpoint [1], [2], [3]. A key question regarding co-expressed genes is whether they are under common transcription regulatory controls (i.e. gene co-regulation), that is, genes that by sharing common *cis*-regulatory elements in their promoters are highly likely controlled by the same transcription factors.

Recent experimental evidence revealed that transcription regulation may reflect also an underlying chromosomal gene positional order [4], [5], [6], [7], [8]. Interestingly, in several eukaryotic organisms, functionally linked and co-transcribed genes have been reported to cluster together in physical proximity unravelling a gene organization with operon-like features, i.e. co-regulation of neighbouring genes [9]. This is supported by the observation that proximity of co-expressed genes does not occur randomly, but rather underlies a selection process involving genes working together in signalling and metabolic pathways [9], [10], [11], [12], [13], [14]. Consistently, experimental investigations revealed that functionally related, co-localized genes show higher probability of being co-expressed than random pairs of genes [15], [16], [17]. The existence of a non-random organization of the genome has been confirmed by the observation that co-localized genes targeted by the same transcription factor are subjected to stronger co-regulation when compared to randomly distributed targets [18]. It has been proposed that the complex eukaryotic transcriptional regulatory mechanism has imposed a significant constraint on gene positional organization along and across eukaryotic chromosomes [19]. Clusters of co-expressed and co-localized genes have been recognized as playing key roles in a number of functional pathways and adaptive responses including organism development, differentiation, disease states and aging.

Although the importance of identifying genes localized in close proximity to each other on the chromosome (gene positional clusters) is widely recognized as a means for unravelling unsuspected functional and transcriptional clues, only few bioinformatics tools and methods are available to support the analysis of large-scale gene datasets [5], [20], [21], [22], [23], [24]. However, they do not allow the investigation of relationships between positional organization of genes within the genome and transcription control. Current methods for the analysis of genome-wide expression data allow to determine which genes show similar increased or decreased expression profiles (i.e. co-expression) in a specific experimental condition, over a number of conditions or throughout a time course. To identify transcriptional networks, co-expressed genes are searched at a later stage for locating common transcription regulatory controls (i.e. co-regulation). Recently, many transcription factor predictor tools have become available. They mostly focus on searching for transcription factor binding sites (TFBS) by using position specific scoring matrices (PSSMs) [25], [26] and by using a comparative genomic approach to identify conserved sequences among homologous promoters, namely phylogenetic footprinting [27], [28], [29], [30], [31]. At present, these approaches still suffer from a high rate of false positive and false negative results. It has been estimated that only 10% of the overall predictions matches to biologically functional binding sites [32]. Integrated approaches using both TFBSs search methods (PSSMs and Phylogenetic footprinting) and gene expression data were recently implemented, leading to a dramatic improvement in specificity while identifying over-represented TFBSs in sets of co-expressed genes [33], [34]. Interestingly, integration of gene expression data has been demonstrated to increase TFBSs prediction specificity, namely enrichment of functional binding sites versus false positives [33]. Another interesting approach [35] attempts at identifying regulatory modules and their cognate regulators from gene expression data using the module network algorithm; although of remarkable importance, the method is characterized by potential limitations, such as: transcription factor post-transcriptional modifications, low regulator expression variation, or the identification of only one regulator out of potentially many.

Here we report on the development of the first standalone software application named CluGene for the identification of co-regulated, co-localized and co-expressed genes.

CluGene integrates gene expression analysis profiling with automated search and identification of co-expressed and co-localized genes while searching for transcriptional regulatory modules. The software combines a number of pre and post processing functionalities together with statistical tools that significantly facilitate and expedite the analysis of gene network co-regulation on a global scale. In addition, CluGene provides a plug-in mechanism so that user-defined functionality can be seamlessly integrated within the software framework, allowing the replication of a multitude of literature algorithms and approaches.

## Materials and Methods

In this section CluGene main functionalities will be described. Further details and more technical details are reported in Text S1. CluGene is available for download from the website of the Bioinformatics Laboratory (BioInfoLab) at the University of Perugia (http://bioinfolab.unipg.it/) and can be freely used for research purposes. CluGene is implemented with the Java Web Start technology and can run on any operating system (Windows, Mac or Linux) where the Java Runtime Environment (JRE) is installed (version 1.6 or higher is required).

### 2.1. Architecture and Basic Principles of Operation of CluGene

A typical CluGene work session revolves around a *project* where gene-related data is managed, analyses are run, and results are generated and visualized. Projects can be saved to files so that any work session can be restored at a later time.

Within a project, gene-related information is collected into one or multiple *gene datasets*. Essentially, a gene dataset is a list of *ENSEMBL geneID* names; however, the dataset is structured so that additional information can be associated to each gene, depending on current analysis needs, such as: the *chromosome* the gene maps to; the genomic localization, its DNA strand orientation, its promoter, one or multiple *transcription factors* matching the promoter and one or multiple *expression levels* as obtained by multiple sources including microarray, EST, qRT-PCR, SAGE, RNA-seq, or other high-throughput sequencing techniques.

A typical work session begins by creating a new project and by importing one or more user-defined lists of geneIDs from CSV or Excel files. The following organisms are currently supported by CluGene: *Anopheles gambiae*, *Culex quinquefasciatus, Aedes aegypti, Drosophila Melanogaster, Homo sapiens, Mus musculus, Caenorhabditis elegans, Saccharomices cerevisiae, Plasmodium falciparum and Plasmodium berghei;* multiple organisms can be managed in a single CluGene project. Additional organisms retrieved from Biomart can be included in CluGene, the process is rather easy, and can be done upon user request to the developers. The possibility of letting the user update the set of organisms within CluGene is being considered for future releases of the software.

In CluGene chromosome names, positional information, promoters and transcription factors are automatically retrieved from online web-services, while expression levels must be loaded into the project from user-defined CSV files. Genomic expression data coming from any online source can be imported into CluGene, as long as it is first formatted into properly structured input files. It is the user’s responsibility to make sure that expression data coming from heterogeneous sources is comparable. This can be achieved by applying several known techniques for normalizing data, e.g. turning expression raw data into fold-changes with respect to given controls.

### 2.2. Functionality Provided by CluGene

CluGene provides multiple options for operating on gene datasets. These can be conveniently organized into the following macro-categories:

#### 2.2.1. Name-based gene dataset processing

This category contains functions aimed at processing lists of genes with set operations (merge, intersect, subtract).

#### 2.2.2. Expression-based gene dataset processing

**Figure 1:**
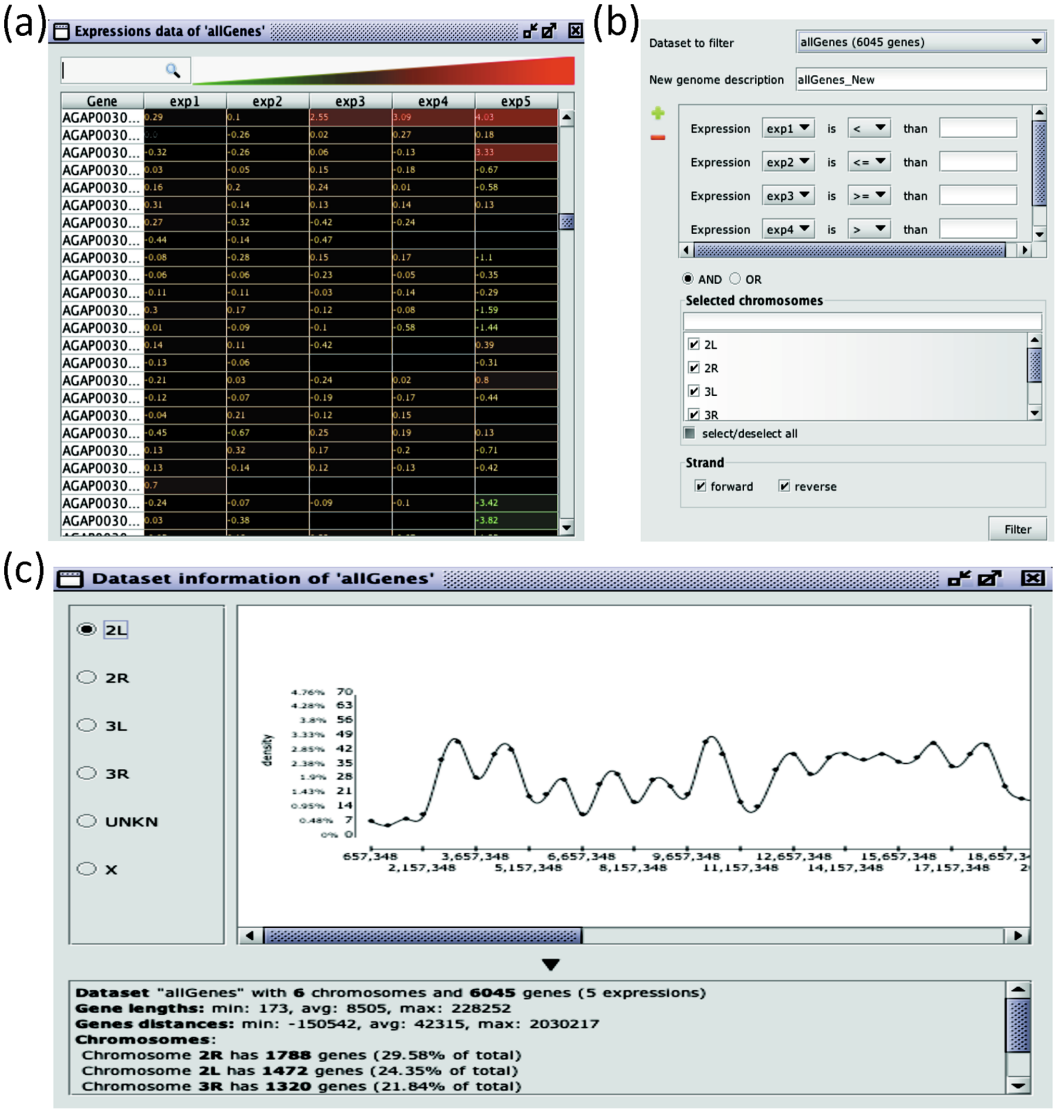
Expression-based gene dataset processing and Position-based gene dataset processing. (**a**). An example visualization of genes with multiple expressions is shown. Expression levels are depicted by the means of different colours (black = 0, gray = not available, from green to red = increased expression values). Data are referring to transcription regulation of sex-biased genes during ontogeny in the malaria vector *Anopheles gambiae* [74]. The 5 different expression datasets are referring to (1^st^, 2^nd^, 3^rd^ larvae stages, pupae and adults). (**b**). An example interactive dialog is shown for gene filtering based on expression level thresholding and Boolean combination of expressions. (**c**). An example interactive window is shown where a chromosome is rendered along with the local gene density plot. Different functions are available for retrieving gene position in the chromosome and their lengths; for retrieving the density of genes along each chromosome, for computing what percentage of a chromosome is represented by a gene dataset; for computing the min, max and average lengths of the genes populating a chromosome; the min, max and average distance between consecutive genes in a chromosome, etc. The dataset considered in this example is the *Anopheles Gambiae* genome.

This set of functions allows the analysis of gene datasets by taking into account expression-related information. The following main types of operation are possible: 1) adding to the current dataset gene expression information loaded from different sources; 2) searching, sorting and visualizing genes based on expression levels; 3) combining multiple expression levels into single scalar values; 3) filtering datasets based on expression thresholds; and 4) comparing datasets based on expression levels. In Figure 1a an illustrative visualization of genes with multiple expressions is shown, where colour is used to indicate expression level. In Figure 1b an illustrative interactive dialog is shown for gene filtering based on expression level thresholding and Boolean combination of expressions.

#### 2.2.3. TF-based gene dataset processing

This category of functions operates with transcription factors (TF) and with TF related information including promoter sequences and transcription factor binding sites retrieved from different online web-services. See Text S1 for detailed information on used web services. For each gene it is possible to identify the position, the number and the identity of the TFBS, and consequently deduce which TFs can interact with them (Figure S1a). The software also allows the reverse search, e.g. by selecting a TF and searching for all the putative promoter sequences containing the corresponding TFBS, which are returned by the software together with their associated recognition frequencies (Figure S1b). Finally, distinct datasets can be compared in terms of predicted TF binding sides (Figure S1c).

#### 2.2.4. Position-based gene dataset processing

Functions are provided for retrieving gene position in the chromosome and gene length; for retrieving the local density of genes along each chromosome, for computing what percentage of a chromosome is represented by a set of genes; for computing the min, max and average lengths of the genes within a chromosome; the min, max and average distance between consecutive genes in a chromosome, etc. In Figure 1c an interactive window is shown where a chromosome is visualized along with the local gene density plot (see later for details) together with statistical information comparing the selected chromosome with the rest of the genome. Datasets can be processed by positional clustering analysis (i.e., identification of groups of genes -clusters- that are in close proximity with each other in the chromosome). This is an example of *co-occurrence analysis* (in this case specifically addressing co-localization) and represents a typical application for which CluGene has been designed.

#### 2.2.5. Multi-attribute gene dataset processing for co-occurrence analysis

This group of functions embodies the main innovative features of CluGene, i.e. the capability of analysing *co-occurrences* (co-localization, co-expression and co-regulation) of genes-related observations for studying transcriptional regulatory networks. These functions revolve around *gene clustering*, i.e. a set of statistical methods for collecting genes into groups (clusters) based on similarity of attributes. The approach followed by CluGene is designed around the following routines:

- *Pre-processing of the gene dataset.* Genes are filtered out using thresholds of expression levels, or by rejecting those genes that either do not contain specific TFBS, or whose corresponding sequences are too short or too far apart, etc. Filters can be customized by taking into account expression, regulation and localization information to better focus on more interesting co-occurrences;
- *Application of positional clustering*. Genes are collected into clusters based on mutual proximity in the chromosome (i.e. co-localization). Clustering can be also driven by additional measures of similarity that take into account expression level and/or TF-related information, in order to obtain groups of genes that are co-localized, co-expressed and co-regulated.
- *Post-processing and analysis of gene clusters. The* clustering results are filtered based on different thresholding criteria that include expression, regulation or position; statistical analyses are performed to determine the significance of the identified clusters in the specific application context.

In the following sections details are provided on positional clustering and analysis of clustering results.

### 2.3. Gene Positional Clustering

Positional clustering plays a central role in the analysis of gene co-occurrences. It operates in different ways, depending on the following customised configurations:

#### 2.3.1. Gene distance metric

Genes are grouped according to similarity assessed by a *distance metric*, which is a function that defines how gene attributes should be accounted for and combined in order to obtain a quantitative measure of similarity.

For all the distance metrics implemented by CluGene, the *starting point* of a gene is the starting point of the first exon of any of the transcripts for that gene; the *end point* is the position of the last exon of all the transcripts that belong to that gene. In Biomart, the coordinates do not change for the −1 strand.

The distance metrics have all constant algorithmic complexity O(1), and are the following:

- *positional (default)*: the distance of two genes is the distance between their starting points in the chromosome; where the starting point coordinates are directly taken from the default Biomart output (which does not make any difference between forward and reverse strands) [5];
- *functional*: the distance of two genes is the distance between the positions of their respective promoters; the promoter is physically located at the starting point of the gene; however, to take into account the behaviour of Biomart which reverses the genes on the −1 strand, the coordinates of the end point of those genes are used as the promoter positions;
- *centre of genes*: the distance of two genes is the distance of their two centres;
- *intragenes*: the distance of two genes is the minimum distance between them (i.e. the distance between the starting point of the second and the ending point of the first)

#### 2.3.2. Gene distance weighting strategy

Weighting strategies can be used to modify the distance metric so that it takes into account also other gene attributes. The weighting strategies implemented by CluGene are the following:

- *uniform weighting:* weights are not used.
- *density based:* this technique tries to capture the concept that a simple gene distance measured in bp (base pairs) may have different functional relevance depending on local gene density. See again Fig. 1c for an example plot of a density function for a chromosome.
- *expression based:* the overall similarity of two genes depends on both their actual proximity on the chromosome, and on the similarity of their expression levels. Two options are available for considering expression similarity. When multiple expressions are available for each gene, and these can be assumed to have a normal distribution within each set, the ANOVA can be used to test the equality of the means among the different groups. The p-value resulting from the test is complemented and used as a weight. Alternatively, a custom weighting function has been designed that works both with multiple and single expression values.
- *transcription factor (TF) based*: promoters recognized by the same TFs significantly affect the overall similarity of two genes. To implement this behaviour, the list of TFs for each gene is retrieved, then the number of shared TFs between the two genes is computed (by intersecting the two lists) and transformed into a weighting factor.
- *user-defined:* a plug-in mechanism implemented within CluGene allows users to define their own weighting functions, which can make use of any available gene information (expression, TFBS similarity, chromosome local density, etc.).

#### 2.3.3. Clustering algorithm

The last parameter that can be selected for gene positional clustering is the type of clustering algorithm. Three options are provided by CluGene:

Clustering by sequential gene processing
K-means clustering
Max-gap Clusters by Multiple Sequence Comparison

In clustering by sequential gene processing, genes are scanned sequentially according to the order they are located in the chromosome. For each gene, its (weighted) distance to the previous is computed. If such distance is below a predefined threshold, then the gene is added to the same cluster of the previous one, otherwise it forms a new cluster. It is important to point out that the threshold is applied to the weighted distance, therefore it must be chosen with care to keep into account also the effects of the weights. This clustering algorithm is the evolution of a previous implementation by the authors [5]. Since genes are ordered by position in the chromosome it is sufficient for the algorithm to check only one pair of adjacent genes at a time, and so the complexity of this algorithm is O(*n*) where *n* is the number of genes, the complexities of the distance metric evaluation and distance weighting function should be added to that in order to obtain a final figure of the overall complexity of the procedure. As a final consideration, it should be noted that one of the combinations available within CluGene, namely positional clustering with density-based weighting, shares many similarities with general-purpose density-based clustering algorithms such as DBSCAN [36] and in particular OPTICS [37]. However, the positional clustering algorithms implemented within CluGene can take full advantage of the fact that distances (and density) are computed over a one-dimensional space (i.e. along the chromosome), which ultimately leads to an algorithmic complexity of O(n), a significant improvement over the typical O(n^2^) of DBSCAN and OPTICS.

The second clustering algorithm available in CluGene is K-means clustering. The *k*-means algorithm is a popular solution for partitioning the items of a dataset into k clusters based on similarity of item attributes. For gene clustering, once a proper distance metric (and weighting function, if required) is defined, the k-means algorithm can be used straightforwardly.

Differently from clustering by sequential gene processing, in this case individual chromosomes are not scanned, and no distance threshold must be manually defined. Typical of k-means algorithms, the operator is asked to input the number of desired clusters *k* that should be located in the dataset. In addition, CluGene provides also an implementation of the method by [38], which is used to predict the optimal number of clusters, which k-means should search for. After having set the number *k* of clusters to be found, the k-means algorithm consists of an iterative process where gene groups are initially formed and increasingly refined. The process starts from an initial assessment regarding the positions of the cluster *k* centroids: at each iteration all the genes are assigned to a cluster, then the centroids are updated according to the updated cluster contents, and the process is repeated until either the centroids have converged to stable positions, or until the maximum number of allowed iterations is reached. The most critical aspect of k-means is in the initial guess of cluster centroids; for this reason, multiple solutions are implemented in CluGene, as listed in the following:

- *random* [39]: k random points are chosen among all valid genes positions;
- *uniform*: k points uniformly distributed on the chromosome are chosen, even if not assigned to valid genes;
- *plusplus* [40]: to spread the k initial centroids away from each other, the first is chosen from a uniform random distribution on gene positions, and each other one is chosen from the remaining positions with probability proportional to its squared distance to the closest existing centroid.

If the plusplus initialization is chosen, the k-means algorithm is guaranteed to find a solution that is O(log k) competitive to the optimal k-means solution.

The third clustering algorithm available in CluGene implements the method known as: Max-gap Clusters by Multiple Sequence Comparison. This algorithm implements the proposal originally from Xu Ling, Xin He and Dong Xin [21]. Our version of algorithm works on pairs of genomes which have orthologous genes in common. Information about orthologous genes is retrieved from Biomart. Given a dataset of one of the supported genomes the user can specify three parameters:

- *Maxgap*: maximum number of non-orthologous genes between two genes in a cluster;
- *Minsize*: minimum size (number of genes) in a cluster;
- *Comparison organism*: the other organism type on which orthologous should be computed.

The algorithm is based on the following steps:

- all orthologous genes, ordered by chromosome position, are computed and genes without an orthologous are removed;
- for each pair of genes in the dataset, they belong to the same cluster if and only if the distance between the two genes computed as the number of non-orthologous genes between them is lower or equal to *maxgap;*
- all clusters with less than *minsize* genes are discarded.

Currently the Maxgap method within CluGene only supports the genomes of *Anopheles Gambiae* and *Drosophila Melanogaster*.

**Figure 2:**
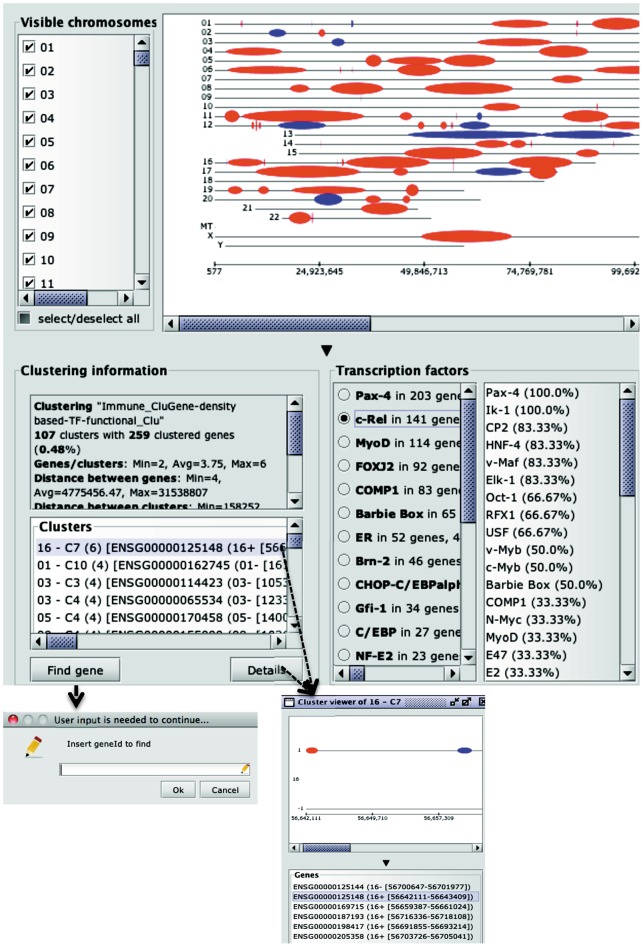
An example of cluster analysis session within CluGene, based on sequential gene processing, functional distance metric and a weighting function combining local density and TF similarity. Clusters are visualized as oval-shapes calculated on the different chromosomes; information concerning the number of clusters and the number of clustered genes is also provided, along with the minimum, average and maximum number of genes present in the clusters, and the minimum, average and maximum distance (bp) between clustered genes and clusters. Detailed information of the distribution of cluster sizes is provided as well. For each cluster, it is possible to visualize the number of contained genes, together with their location and the list of TFs with their associated frequencies. Besides, it is possible to visualize, for entire clustered dataset, the calculated TFs together with associated frequencies. For each TF it is possible to identify the number of recognized target genes and the relative number of clusters. Once a TF is selected, the clusters positive for the specified TF are interactively highlighted in red whereas negative clusters remain blue (default colour).

An exemplifying cluster analysis session within CluGene is illustrated in Figure 2, based on sequential gene processing, functional distance metric and a weighting function combining local density and TF similarity.

### 2.4. Analysis of Statistical Significance of Gene Clusters

CluGene offers functions for post-processing the clustering results in order to highlight significant aspects. Two main alternatives are provided: *comparison to a random set*, and *neighbourhood model and clustering statistics.*

#### 2.4.1. Comparison to a random set

The clustering results obtained from the available dataset are compared with the results of the same clustering process applied to a random set of genes. The goal is to see whether the clustering of the available genes produces a number of clusters which is significantly different than what could be obtained by aggregating random genes. To perform this analysis, CluGene needs to produce an alternative set containing a random set of genes, as large as the original set (size: n). The random set is created by randomly extracting n genes from a user-selected set (size>n). No constraints are imposed on the choice of such set, which may or may not even contain some or all the genes of the dataset being scrutinized. Once the random set is ready, CluGene runs the same clustering analysis that was performed onto the original dataset and the entire process (selection of random genes and clustering) can be repeated multiple times. Once the process is finished, the results from clustering of random genes are aggregated and compared with the “real” clustering results. The comparison is done through hypothesis testing: CluGene provides the one-sample t-Student test to compare number of clusters. Ideally, for the “real” clustering to be significant, a different number of clusters should be generated in the “real” case with respect to the random case, and such difference would be statistically significant (low p-value). In its current implementation, CluGene can only assess if the number of clusters in the test case is significantly larger than the random case, and if such difference is statistically significant (one-tailed, one-sample t-test). This approach may be limited, and may fail to flag as interesting those cases where the opposite happens (e.g. gene sets belonging to the same functional pathway may be more likely be localized within the same cluster). Future CluGene implementations will consider the adoption of a two-tailed test to assess significant differences, regardless of the sign. Also, a more complete offering is planned for future implementations of CluGene, which includes non-parametric tests, as equally viable and sometimes preferable alternatives to parametric tests such as the one-sample t-test. In the current implementation, any other test which is not the one-sample t-test can still be performed, but this requires manually exporting the CluGene results into separate software applications.

#### 2.4.2. Neighbourhood model and clustering statistics

CluGene implements an additional algorithm to test statistical significance of the computed clusters, as originally defined by Li et al [23]. In a sense, this is similar to the previous comparison to a random set. For each chromosome, the mean number of adjacent genes in a random arrangement and the standard deviation is computed.

## Results

In order to study the capabilities of CluGene we performed two different analyses (case studies) testing the different clustering algorithms and analysis tools of CluGene. Organisms of the genuses *Homo* and *Plasmodium* were used in the validation process.

### 3.1. Case Study 1: Co-localization of the Binding Sites of Oct-1, c-REL, NF-KappaB, IK-1, BSAP and CP2

Specific transcription factors contribute to immunoglobulin and other relevant genes expression crucial for B cell development and function. They include octamer-binding transcription factor 2 (Oct-2), B cell Oct-binding protein 1 (BOB.1), B cell–specific activator protein (BSAP), BCL-6, MUM1/IRF4, PU.1 (also known as Spi-1), Oct-1, c-REL, NF-KAPPAB, IK1 and CP2 [41], [42], [43], [44], [45], [46], [47], [48], [49], [50], [51], [52], [53], [54], [55], [56], [57]. Unbalance in the synthesis of these transcription factors or the presence of mutations can lead to suboptimal activation of immunoglobulin or of other relevant gene promoters in different B cell tumors [43], [58], [59], [60] [61], [62], [63]. B-cell transcription factors show diverse expression patterns in different lymphomas (i.e. Hodgkin lymphoma (CHL) from diffuse large B-cell lymphoma (DLBCL) [41] or in non–germinal center small B-cell lymphomas [57] (a heterogeneous group of non-Hodgkin lymphomas).

Being able to carry out a detailed stratification analysis based in the identification of individual combinations of transcription factors would be of great clinical value in terms of diagnosis, prognosis and response to therapy.

To test the capability of CluGene in disclosing networks of co-regulated genes, the human genome data and whole genome arrays from diffuse large B-cell lymphoma (DBLCL) specimens were considered.

#### 3.1.1. Datasets for the diffuse large B-cell lymphoma

In previous work [64], a combined approach of transcriptional profiling and gene set enrichment analysis had highlighted the existence of three distinct DLBCL sets containing a significant number of functionally related genes, referred to as: *oxidative phosphorylation (*OXPHOS), *B-cell receptor/proliferation* (BCR/proliferation) and *host response* (HR) (downloadable from http://www.broadinstitute.org/cgi-bin/cancer/datasets.cgi). The human gene IDs in the Ensembl format were obtained using Clone/Gene ID converter [65].

#### 3.1.2. Identification of the main TFBS motifs

We first investigated which of the annotated B cell TFBS motifs were represented in the promoter sequences of the three gene subsets. The putative promoter regions needed for the functional distance metric were retrieved directly from Biomart and correspond to the 1000 bp flanking the 5′ end of each gene. According to the Match™ prediction functionality provided by CluGene the binding sites for transcription factors Oct-1, NF-KAPPA-B, IK-1, c-REL, BSAP and CP2 occurred in the promoters of the three subsets with different frequencies (Figure S2a). In particular, in the OXPHOS subset, the Oct-1 TF showed the highest number of binding sites. For assessing whether the presence of such a high number of binding sites had a functional significance, we compared the result with the number of Oct-1 TFBS found in a dataset of genes randomly extracted from the human genome. The random gene set (same size of the original dataset) was generated through the functions available within CluGene, and analyzed with the same Match™ prediction function. All the results were exported from CluGene in order to apply the one-sample Student’s t-test provided by a commercial statistical software application (SPSS) to test for significant differences between the study and random datasets. The test showed that the higher number of Oct-1 TFBS found for the real dataset was significantly different from the amount of binding sites found for the random set (Figure S2b).

#### 3.1.3. Dataset clustering behavior under the application of different distance metrics

To begin analyzing the relationships between co-regulation and co-localization, a first investigation was aimed at identifying interesting clustering behaviors of the datasets subjected to sequential clustering, under the application of three main distance metrics implemented within CluGene:

- first metric: positional with no weights;
- second metric: functional (promoter distance), weighted by local density;
- third metric: functional (promoter distance), weighted by local density and TF similarity.

**Figure 3:**
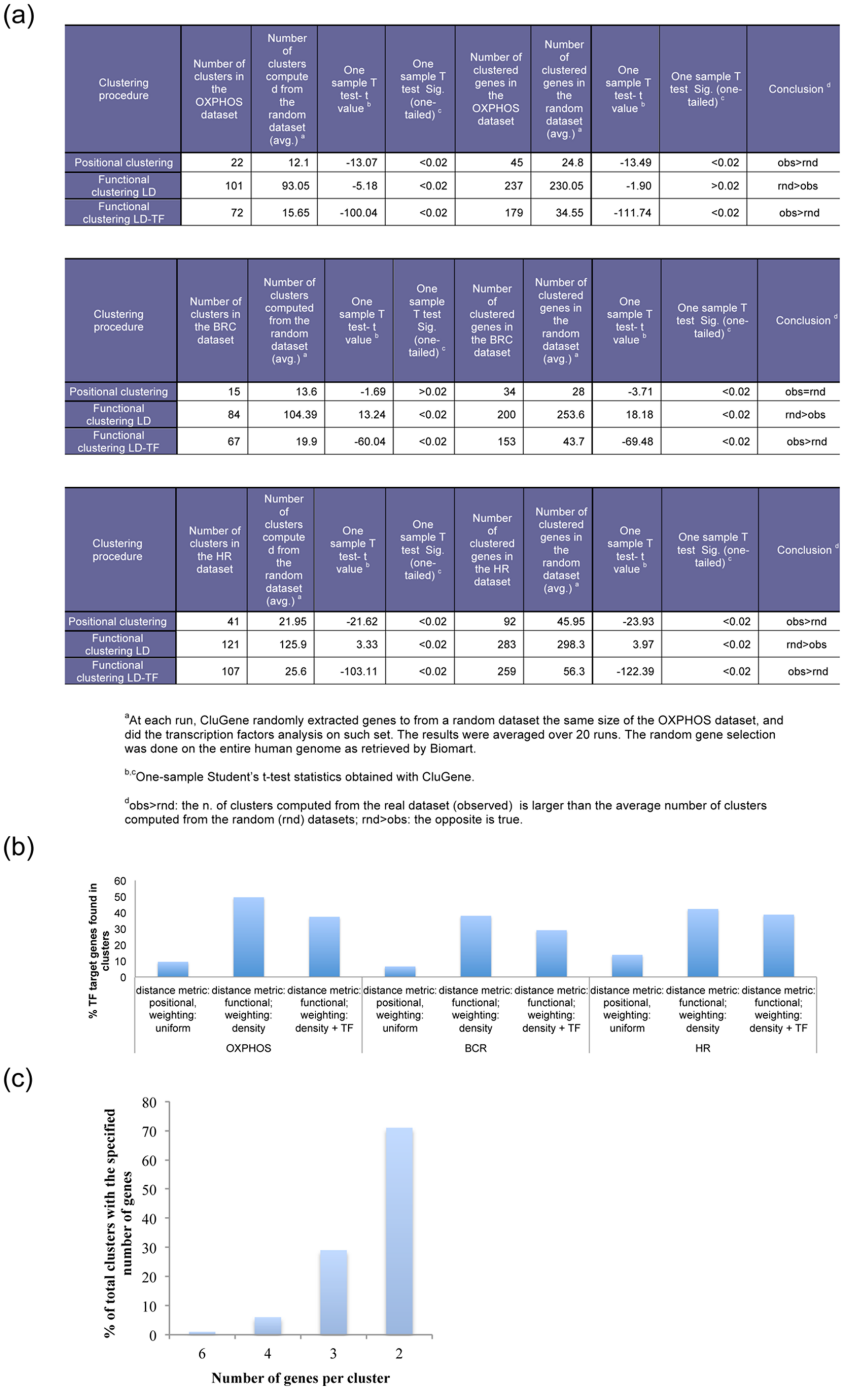
Different clustering behaviours of the DLBCL subsets subjected to sequential clustering and different distance metrics. (**a**). The one sample t-test was used to validate the statistical significance of the number of clusters found in the test datasets, vs. number of clusters found in random datasets. (**b**). % target genes found in clusters with: simple positional distance metric, functional with local density and functional with local density and TF-similarity. (**c**) A histogram showing the frequency distribution of clusters is shown.

A threshold value of 83,000 bp [66] on the distance metric was adopted as the rule for cluster formation for all strategies. The number of identified clusters was tested for statistical significance with one-sample t-test (through a function available within CluGene) against a random set of genes. Since the population may be nonnormal the power of the one-sample t-test may be slightly reduced, albeit not that much, given the sample size. The third metric generated a significantly larger number of clusters compared to the random set (Figure 3a), this applied consistently for all three datasets. Also, CluGene post-process analysis of the clusters produced with the third metric showed that a high percentage of genes targeted by the selected list of TFs are co-localized within the genome (Figure 3b).

The application of the third metric highlighted also that in the HR subset there is the highest percentage of genes (45.17%), which can be found in large clusters (defined as clusters containing three or more genes). A histogram showing the frequency distribution of clusters is shown in figure 3c. This preliminary result may indicate that the HR subset is under transcriptional co-regulation by the same set of TFs.

**Figure 4:**
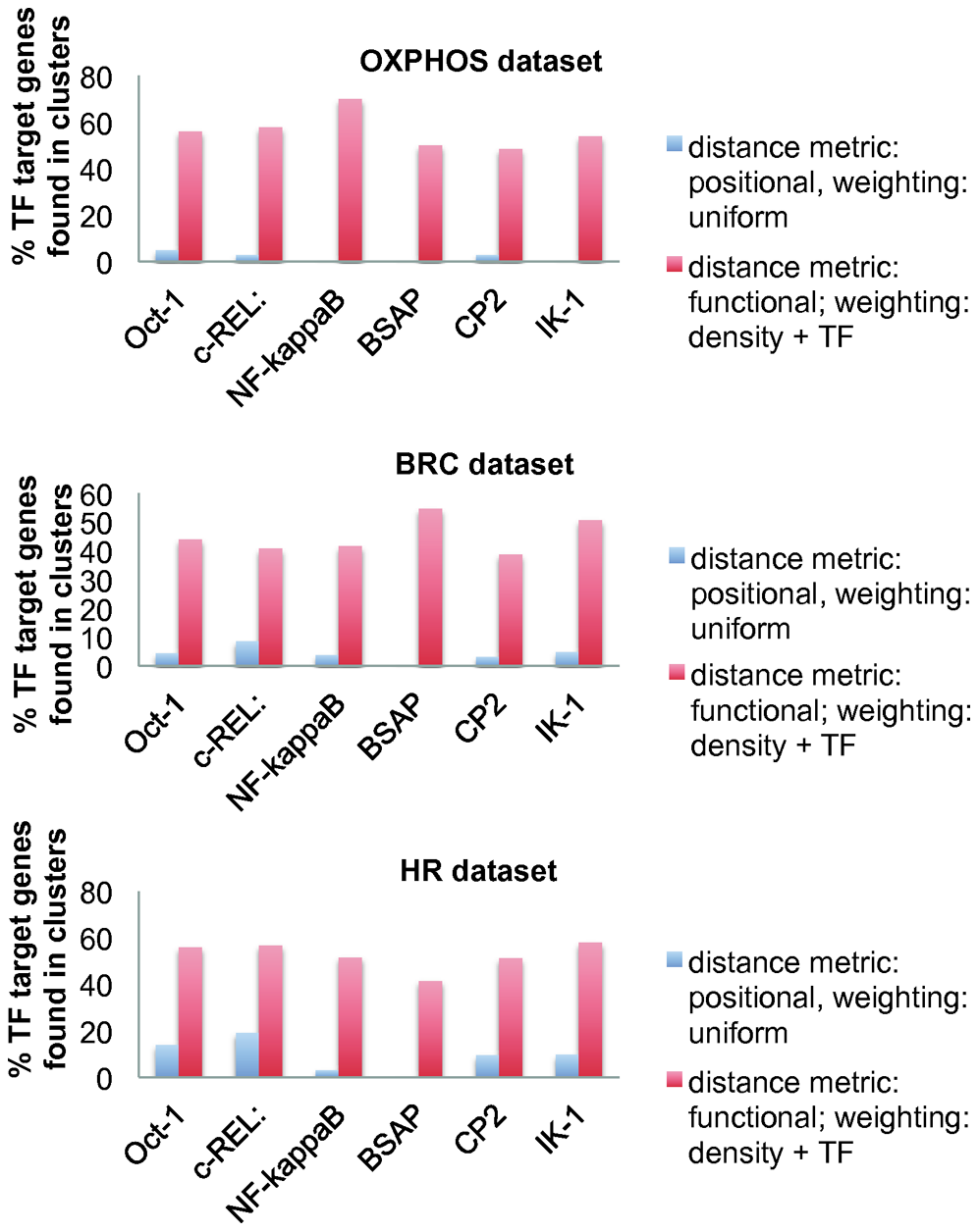
Percentage of of co-localized positive annotated B-cell transcription factors. Percentage of Oct-1, NF-KAPPA B, IK-1, c-REL, BSAP and CP2 target genes found in clusters obtained with the functional distance metric weighted by local gene density and TF similarity and with the simple positional distance metric, out of the total amount of recognized TF target genes.

To better investigate the benefits of utilising the third metric (promoter distance, weighted by local density and TF similarity), we considered the identified B cell transcription factors (Oct-1, NF-KAPPA B, IK-1, c-REL, BSAP and CP2) and the co-localization of their cognate target genes along the genome. Notably, a higher amount of co-localized positive annotated B-cell transcription factors target genes was identified with the third metric (Figure 4) compared to the application of simple positional clustering (first metric). In particular, with slight variations depending on the subset, the percentage of genes, originally predicted as targets for the TFs and found in clusters obtained with the third metric reached 70%, while the same percentage obtained through simple positional clustering was not higher than 18.8%. This result suggests that DLBCL co-regulated genes are physically associated in clusters. Such clusters are more subtly defined than what can be detected by means of proximity alone. The percentages of TF target genes found in the clusters are shown in Figure S3.

#### 3.1.4. Investigations on the co-localization of the binding sites of the selected TFs

**Figure 5:**
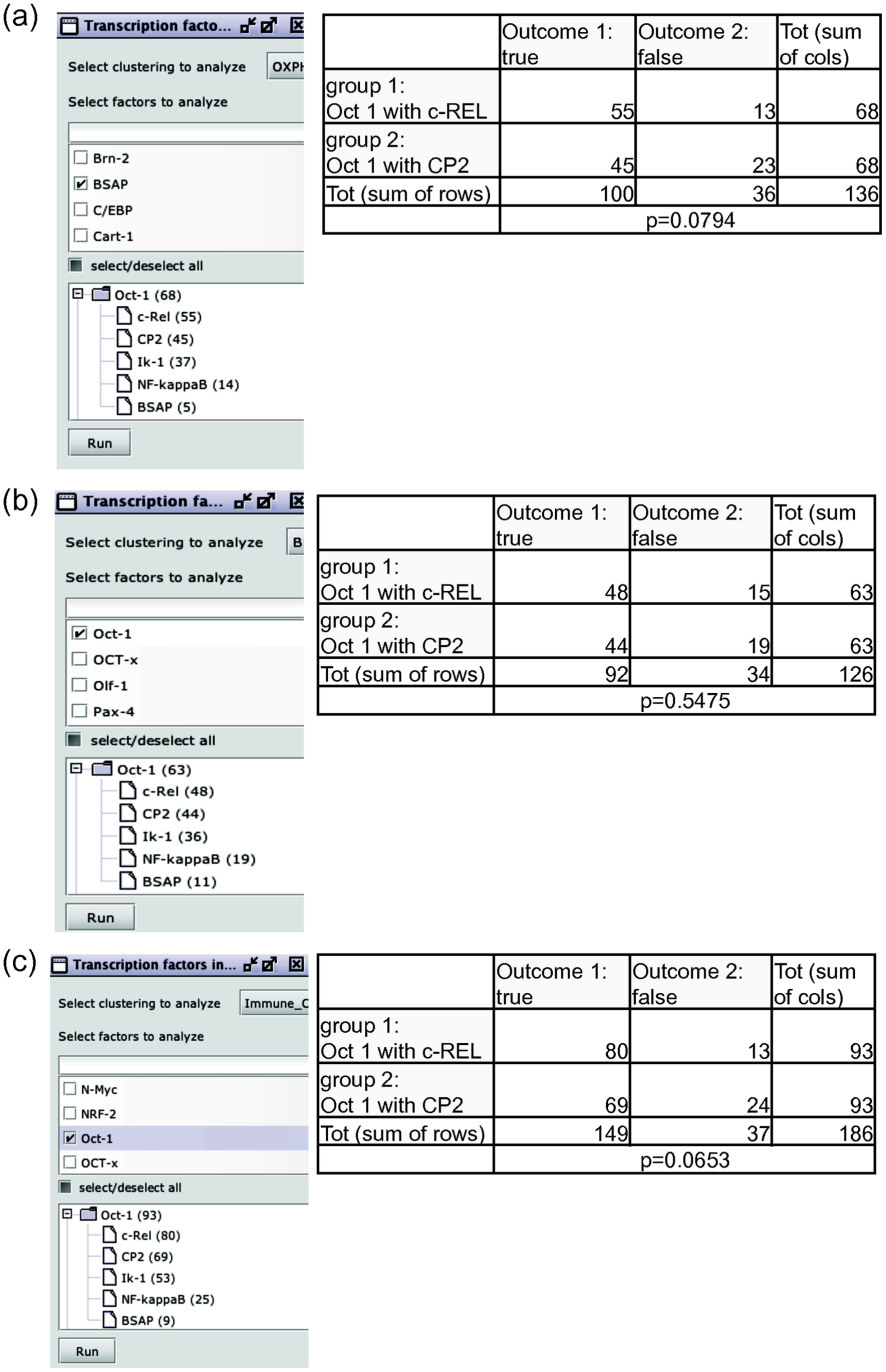
Analysis of co-operative regulatory effect of Oct-1, c-REL, NF-KappaB, IK-1, BSAP and CP2 in shared DLBCL genomic sites. Co-operative regulatory regions in target genes were identified using the functional distance metric weighted by local density and TF similarity. The panels refer to an investigation aimed at identifying TFs appearing more often together in clusters. The values in the contingency tables (right panels) are the frequencies of clusters featuring either both TFs of a given pair (true), or only one of them (false). The tables contain also the p-values of the Fisher’s exact test, indicating whether there is some statistically significant difference between the co-occurrences of TF pairs within clusters. Panels refer specifically to the (a). Oxphos; (b). BRC and (c). HR subsets. Only statistically significant results are shown. These results emphasize the role of c-REL, CP2 and Oct-1 association in shared diffuse large B-cell lymphoma regulatory regions.

We also investigated whether Oct-1, c-REL, NF-KappaB, IK-1, BSAP and CP2 co-localize themselves within the same genomic sites, which may imply a possible co-operative regulatory effect. Again by using the functionality provided within CluGene, we investigated if specific pairs of TFs would co-occur more frequently than others within clusters. As a starting point, the clustering results obtained with the more promising third distance metric were considered. The analysis specifically highlighted a statistically more frequent co-occurrence of Oct-1 with c-REL and CP2 as judged by the contingency tables created to compare any two pairs in terms of number of clusters featuring them (Figure 5); contingency tables were created to compare any two pairs of genes in terms of number of clusters featuring them, to see whether specific pairs of TFs appeared more often within the same clusters. The contingency tables were created using the GraphPad QuickCalcs Web site (http://www.graphpad.com/quickcalcs/Contingency1.cfm). However the test for statistical significance of this result, which was based on running within Clugene the same type of analysis on clusters obtained from random genes, did not pass. While it is yet unknown if the observed associations might ultimately provide a contribution to the understanding of B-cell lymphomas pathogenesis, it is interesting to note that the identified co-occurrences include genes which have been already recognized as being part of the same functional pathways in large B-cell lymphoma, and this finding applies to all three different DLBCL subsets.

#### 3.1.5. Identification of specific diagnostic markers of DLBCL

**Figure 6:**
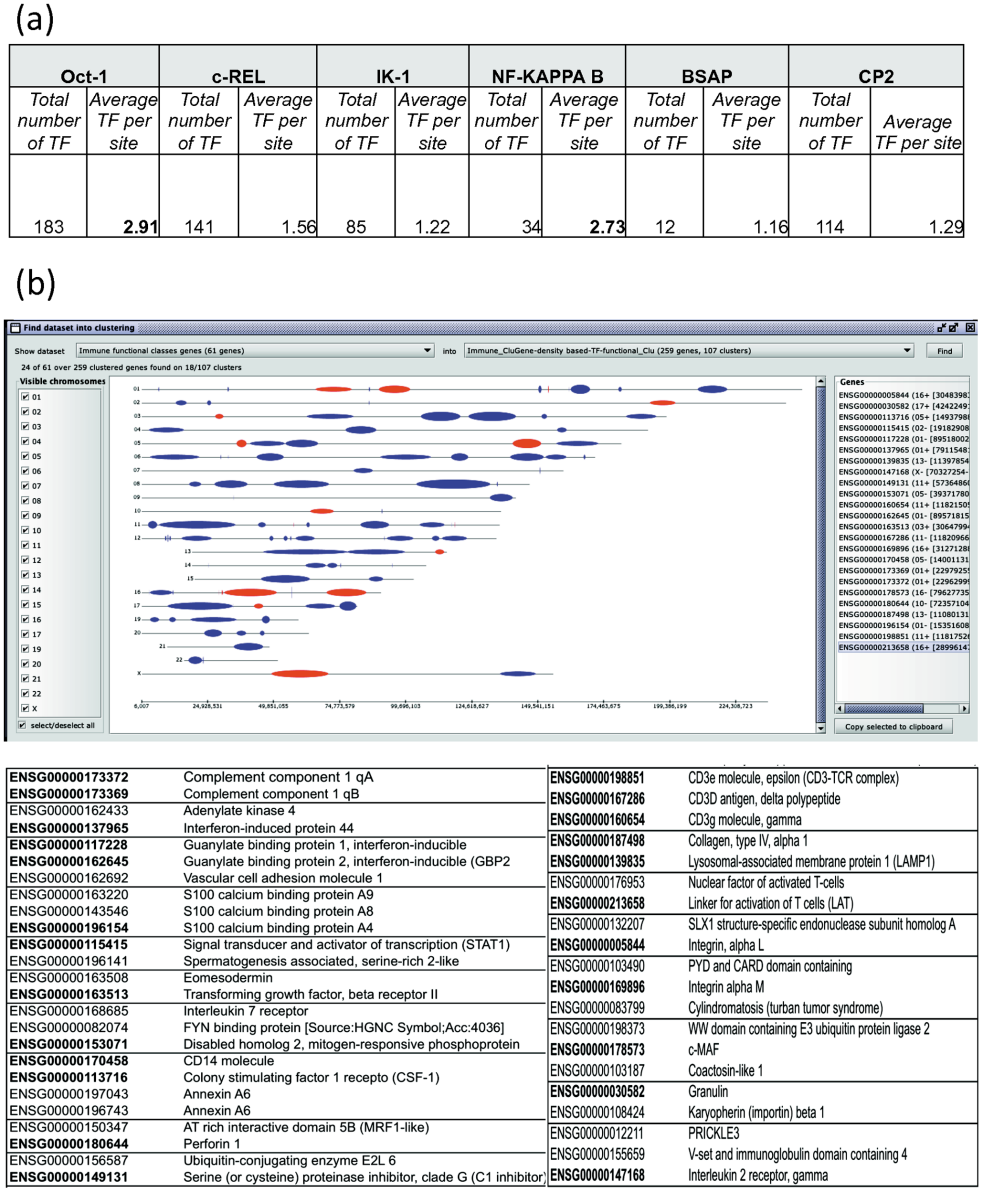
Characterization of the immune/inflammatory response genes of the HR subset. (**a**). Total and average number of TFBSs present in the promoters of genes expressed in the HR subset. Frequencies were calculated on the genes clustered using the functional distance metric weighted by local density and TF similarity. In bold are shown TFs (Oct-1 and NF-KAPPA B) with an average number value exceeding 2 sites/promoter. (**b**). Analysis of localization within clusters of HR genes having the signature of immune/inflammatory response. The *Show dataset into clusters* utility of CluGene (top panel), allows to identify which clusters (red) out of the total (blue) contain a specified list of genes (immune annotated genes). The list of genes contained in clusters and their location is given on the right part of the panel. The tables (bottom) include the identified clustered immunity genes (bold), their function and the size and what other genes are grouped within the same cluster.

Additional investigations were made on the datasets with the ultimate aim of providing support to the studies addressing the identification of specific diagnostic markers of DLBCL. As pointed out by Monti and co-authors [64], the HR subset represents an interesting source of potential treatment targets as a consequence of the role of the immune response featuring this DBLCL subset. To this aim we have applied the same distance metric illustrated above (promoter-based distance metric with weights based on local density and TF similarity) to the genes of the HR subset, and then we used CluGene functions to compute the average number of TFBSs located within the promoters of the clustered genes. Interestingly, this analysis indicated that both Oct-1 and NF-Kappa B had -on average- more than two regulatory cognate site regions per promoter (Figure 6a). Then, by investigating the percentage of clustered HR genes, which had also the signature of immune/inflammatory response, we were able to estimate the number of immunity-annotated genes that mapped to proximal chromosomal locations (figure 6b). Interestingly almost 40% of the immune markers appeared to be co-localized.

Finally, we examined whether co-regulated and co-localized genes in the HR subset would also show similar expression levels, which may indicate similar transcriptional activity.

Previous literature work had outlined that co-localized, TF target genes are subjected to tighter co-regulation compared to isolated genes, and genes being co-regulated by common TFs tend to have a similar expression profile [18], [35], [67].

For each DLBCL transcription factor identified as illustrated above, we first separated co-localized genes being recognized by that TF (test group) from those that although being co-localized were not recognized by it (control group). Since some of the expression profiles for the two groups were nonnormal, for each TF we compared the gene expression profiles of the two groups using the Mann-Whitney U test at a threshold of p<0.05 (SPSS Statistics v.17.0).

**Figure 7:**
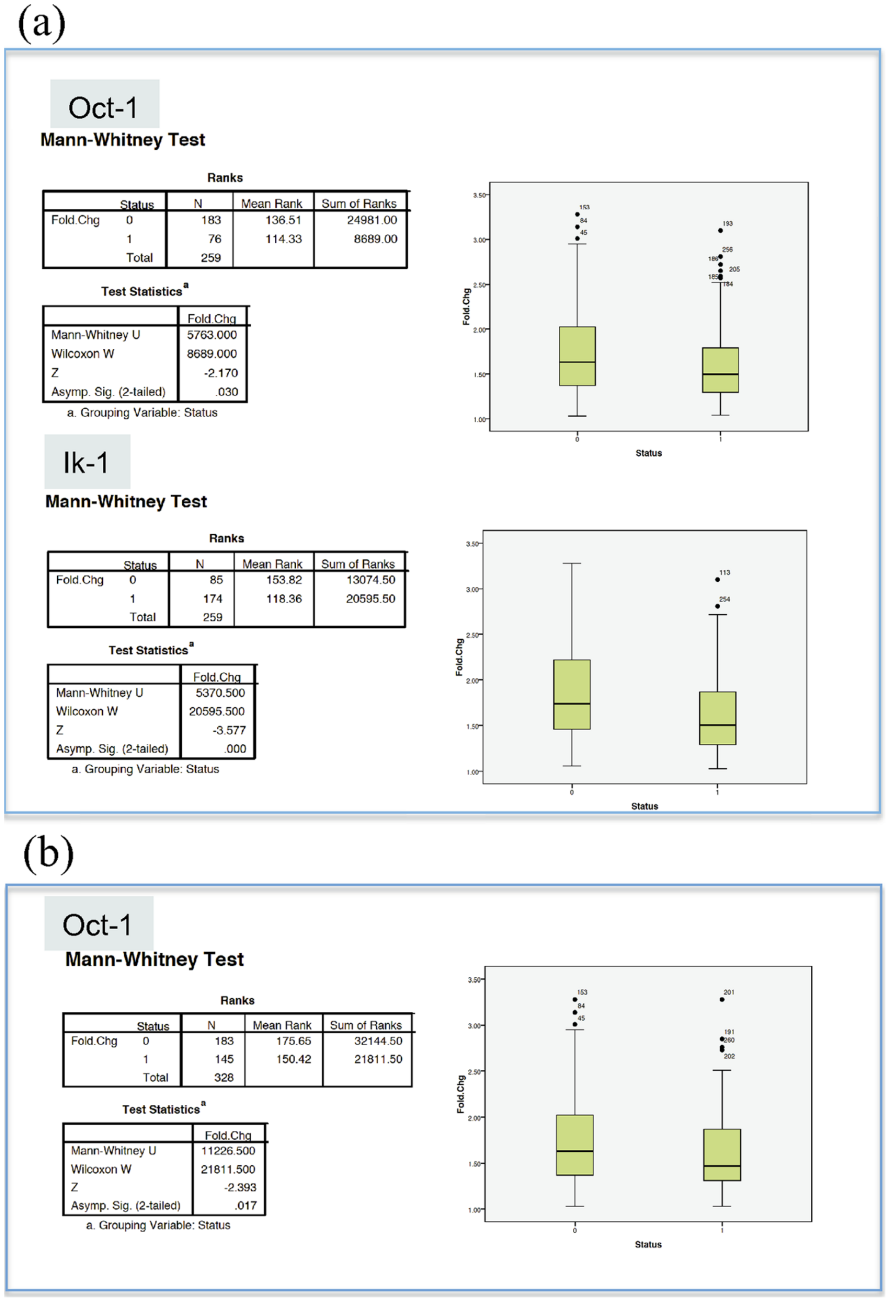
Co-localization of TF target genes positively affects co-regulation. (**a**). Comparison of expression profiles of genes showing both co-localization patterns and recognition patterns by a specific DLBCL annotated transcription factor (Status = 0) from those that although being co-localized were not recognized by the specified transcription factor (Status = 1). The comparison of the gene expression profiles between the two groups was done using the Mann-Whitney U test at a threshold of p<0.05 and results were illustrated as boxplot. Only TF co-localized target genes differing in their expression patterns with respect to those not recognized by the specific TFs have been detailed (Oct-1 and IK-1). (**b**). Both co-regulation by a common TF and co-localization patterns determine co-expression of TF target genes. The expression profiles of Oct-1 co-localized target genes (Status = 0) were compared to those that although recognized by Oct-1did not cluster together (Status = 1); notably Oct-1 co-localized target genes differed in their expression from those that did not cluster as shown by the Mann-Whitney U test at a threshold of p<0.05.

Notably, co-localized genes containing both Oct-1 and IK-1 binding sites showed statistically significant difference in the expression patterns (higher expressions) when compared to the control group (Mann-Whitney U test p_Oct-1_ = 0.03; Mann-Whitney U test p_IK-1_<0.01) (Figure 7a). This behaviour was not shared by the other TFs (NF-KAPPAB, c-REL, BSAP and CP2), which did not show expression differences between the two groups.

To investigate whether gene co-expression was a consequence of either sharing specific transcription factor binding sites; or being in chromosomal proximity, we compared the expression profiles of Oct-1 target genes that showed co-localization patterns with those that did not show such pattern by using the Mann-Whitney U test at a threshold of p<0.05. Notably, Oct-1 co-localized target genes differed in their expression –higher expression- from those that were not co-localized (Mann-Whitney U test p = 0.017) (Figure 7b).

Taken all together, these results suggest that co-localized genes are more likely to be co-regulated than isolated genes. As the observed correspondence between co-regulation and co-localization was not shared by all examined TFs, we expect that not all annotated TFs have a stringent or relevant role in DLBCL or either that the changes in expression patterns are not sufficient to be detected.

As a side note, it should be pointed out that another valid non-parametric test that could have been adopted for the investigations illustrated above is the Kolmogorov-Smirnov (KS) test. While the KS test is capable of detecting a wider array of differences in the shape and localization of two distributions, in this specific case, we were only interested in simple comparisons of the means of expression values (i.e. figuring out whether one group would have significantly higher expression patterns than the other), which is why the Mann-Whitney U test was adopted.

### 3.2. Case Study 2: Specificity of Genes Containing the Host Targeting (HT) Motif in *P. Falciparum*

To investigate the capabilities of CluGene clustering algorithms, we first analysed the entire genome of *Plasmodium Falciparum* by considering genes having different cellular localization attributes. Genes exhibiting different localization attributes were downloaded from PlasmoDB website (http://plasmodb.org/plasmo/), using the following queries: (i) Genes with signal peptide [68]: genes that are predicted with the of signal peptide cleavage sites in amino acid sequences, predictions are made with the SignalP program; (ii) Genes with transmembrane domains [69]: genes with at least a transmembrane helix in encoded proteins, prediction are made with the TMHMM2 program; (iii) Apicoplast Genes [70]: genes targeted to the apicoplast membrane, a derived non-photosynthetic plastid found in most Apicomplexa; (iv) Genes with Host-targeting (HT) motif [71]: genes with sequence motifs identified by Hiller and co-workers [71] as being associated with proteins secreted to the human erythrocyte. Proteins secreted to the host erythrocyte represent important modulators of the antigenic and adhesive modifications in the infected erythrocytes [71].

To compare results from different research analyses and from different Plasmodial species, we also considered an additional dataset [72] containing genes of *P. Falciparum* and *Plasmodium Berghei* exhibiting the HT motif.

First, all downloaded dataset were imported into CluGene system and then CluGene’s visualization tools were used to compute the gene densities present across all chromosomes in the different datasets. All datasets were then clustered using the default options: positional clustering algorithm with constant threshold, using a positional gene distance strategy (gene distance defined as the distance between genes’ starts) and subsequently the obtained clustered datasets were compared to each other. From the output of CluGene analysis for PlasmoDB datasets (genes containing signal peptide, genes containing transmembrane domains, genes containing the Apicoplast internalization signal and *P. Falciparum* genes containing the HT motif) we found that gene density is almost linear for all chromosomes and that genes are distributed almost uniformly on all chromosomes with the exception of genes containing the HT motif (see Figure S4 (a) for density and distribution examples). The gene density of genes containing the HT motif is always higher at chromosome ends and almost zero in the centre of chromosomes on all chromosomes. HT genes, in fact, are mostly distributed at chromosome ends (see Figure S4 (b)), when computing a positional clustering with CluGene we observed a co-localization of 192 clustered genes in 36 clusters (90% of the total number of genes, see Figure S4 (c)).

The significance of this clustering was assessed against generated random clusters that resulted in having at most 50 genes in 30 clusters (just 23% of the total number of genes).

When we analysed the *P. Falciparum* and *P. Berghei* HT datasets described by Sargeant [72], for comparison, we found a similar clustering behaviour for both *P. Falciparum* and *P. Berghei* gene datasets (data not shown). CluGene clustering features and visualization tools helped in identifying this inter-species clustering behaviour in a very simple way.

## Discussion

The importance of adding positional information to the data generated by high-throughput techniques, such as histone marks, chip-seq, and DNAseI hypersensitivity, is already recognized. Adding gene localization information could also provide benefit to projects such as the Encyclopedia of DNA Elements (ENCODE) project [73].

Despite the benefits of positional information, obtaining a comprehensive picture of transcriptional regulatory networks is still beyond our reach. Additional insight can be gained by studying co-occurrences, i.e. events involving activation/deactivation of clusters of genes that not only are localized within the same confined regions on the chromosomes, but also respond to the same transcription factor families, and are similarly expressed.

No currently available bioinformatics tool allows for multi-attribute gene searches where co-localization, co-expression and co-regulation can be considered at the same time.

CluGene offers such possibility for the first time, and provides flexible integration mechanisms to assign varying relative importance to co-localization, co-expression and co-regulation to accommodate the needs of many types of analysis processes.

It is particularly important to stress out the *concurrency* of the search criteria (localization, expression and regulation) allowed by CluGene. A possible alternative would consist in running multi-step searches that proceed by further refinements: e.g. a first positional clustering to identify groups of genes that are located close to each other, followed by filtering on expression levels, and again followed by searching for common TFBSs in order to identify those subsets of co-localized, co-expressed and co-regulated genes. It goes without saying that such a sequential approach would greatly limit the amount of promising genes that in the end survive the bioinformatics selection process, each step potentially eliminating promising candidates only because of the order the selection operations were performed. On the contrary, with CluGene, such steps can be integrated so that all the criteria can be applied at the same time, and with weights, so that the relative importance of each can be controlled.

The case studies have shown that there may not necessarily be a unique analysis protocol that works equally well for any type of problem; which means that in general, for a new problem, many pathways may be explored involving numerous combinations of multi-attribute clustering and weighting strategies before important novel insight can be gained, and emerging behaviour can be detected. This brings to the table the second main distinguishing feature of CluGene, i.e., the software being an interactive framework where different solutions can be rapidly explored and compared. Furthermore, through the plug-in mechanism, CluGene allows for user-defined algorithms and procedures to be integrated within the framework to expand its functionalities, reaching an optimal compromise between the accessibility of a user-friendly solution and the flexibility of a programming environment.

The types of investigations that may be run with CluGene are numerous, ranging from simpler searches trying to determine if a specific TF regulates nearby genes, or analysing the tendency of a TF to preferentially regulate genes located on one or a few specific chromosomes or chromosome regions, to more complex analysis tasks, such as determining whether TF regulatory mechanisms have lead to evolutionary constraints favouring the formation of specific positional clusters of target genes, and whether clusters of co-regulated genes show some tendency of producing similar expression levels as well.

A limitation of CluGene concerning TF analysis is that it currently supports only the analysis of binding sites in promoters; in future releases binding information will be searchable also in other locations across the genome, such as enhancers, introns, etc. (e.g. analysis of ChIP-Seq data).

Moreover, despite the capacity of CluGene to provide novel viewpoints for understanding transcription regulation, it still fails to provide insight on higher-order phenomena. Specifically, the chromatin remodelling events leading to inactive and active states and the three-dimensional, spatial organization of the chromosomes within the nucleus (i.e. chromosome territories) are currently beyond the analysis capabilities of CluGene. Our method typically identifies only the first level of transcription regulation: further applications will certainly need to take into consideration higher-order transcription regulation in order to provide more tools for understanding transcription regulatory mechanisms.

Nevertheless, it is our belief that as genome-wide transcriptional analysis and in general genome high-throughput experimental technologies will keep on providing a growing amount of information on different organisms, the application of software tools such as CluGene will become increasingly more important.

The availability of techniques that allow for the identification of relationships between gene properties and regulation phenomena will reveal itself as a fundamental prerequisite for the development of original and performing search tools to shed some more light on transcriptional regulatory networks.

## Supporting Information

Figure S1 **TF-based gene dataset processing.** (**a**). For each gene present within a dataset (left panel) it is possible to identify number and types of TFBS, and consequently, what TFs can recognize them. (**b**). It is also possible by selecting a specific TF to search for all genes being recognized by it together with associated frequencies, a list of all TFs recognizing each gene in dataset is provided as well. (**c**). Distinct datasets can be compared in terms of predicted TFBs. The comparison can be ordered as a function of difference or relevance. TFs are coloured differently in the compared datasets depending on the outcome: present at an higher extent (green), present at a lower extent (red) and absent (black).
(TIF)

Figure S2 **Transcription factor binding site motifs over-represented in sequences from the three DLBCL subsets (BRC, HR and OXPHOS).** (**a**). List of TFs with associated frequencies for each DLBCL subset according to the MatchTM predictor embedded in CluGene. Annotated B cell transcription factors are boxed. TFs are coloured differently in the compared datasets depending on the outcome: Higher transcription factors binding site motifs percentages are coloured green whereas lower percentage are marked in red, absent TFBSs are coloured balck. The last column shows the maximum difference values amongst TF frequencies within the three subsets. (**b**). Statistical significance assessment for the results obtained with selected TFs in the OXPHOS dataset with respect to a random set of genes. Transcription factors results on the OXPHOS dataset were compared with average transcription results on 10 datasets of randomly selected genes. Ten random sets of genes were extracted form the human genome, with a number of genes same as OXPHOS. The One sample student’s t-test (SPSS) was used for the analysis.
(TIF)

Figure S3 **Percentages of genes that are TF targets.** Number of TF target genes out of the total number of genes present within the three DLBCL subsets. A comparison between the number of TF target genes in the three DLBCL subsets and different clustering strategies output is presented. Gene clustering was performed using both the *un-weighted positional clustering* (Positional clustering) followed by TF prediction and the *functional clustering weighted by local density and TF similarity.* For the positional and functional clustering cases, the percentage refers to genes found in clusters.
(TIF)

Figure S4 **Localization of genes containing the HT motif in** ***P. falciparum*** **.** The gene density and the cluster of genes containing specific localization attributes were predicted throughout the *Plasmodium Falciparum* genome using different source datasets. (a) Calculation of the gene density of genes containing the signal peptide (PlasmoDB) as localization attribute, a total of 1216 genes were considered for the analysis. The left-hand panel shows the distribution of the gene density on chromosome 5 as an example, while the right-hand panel shows the localization of genes containing the signal peptide on all chromosomes. The relative position of the individual genes (blue bars) are shown along the chromosomes (horizontal lines). The positions of the genes along the chromosomes are also indicated with a label that represents the position (in kbp). (b) Calculation of the gene density of *P. falciparum* genes containing the HT localization motif (PlasmoDB), a total of 214 genes were considered for the analysis, the left-hand panel shows the calculated gene density on chromosome 5 as an example, while the right-hand panel shows the localization of the genes containing the HT motif along the different chromosomes: genes tend to locate mostly at the end of the chromosomes. (c) Clustering analysis of the dataset containing the genes with the HT motif (same dataset as in (b)). The relative position and the size of individual genes clusters (blue-oval shape) are shown along the chromosomes (horizontal lines). The size of each cluster is proportional to its population size. Each cluster is also flagged with a label that indicates the position (in kbp) of the first gene. A total of 192 genes in 36 clusters were found out of the total 214. Gene clustering was performed using default positional clustering options.
(TIF)

Text S1 **Supplementary information about the CluGene software.** Download details; system requirements; third-party libraries used by CluGene; genome and transcription factor online databases accessible from within the software; technical details on the clustering algorithms available in CluGene.
(DOCX)
