# Supplementary figures and images for: CluGene: A Bioinformatics Framework for the Identification of Co-Localized, Co-Expressed and Co-Regulated Genes Aimed at the Investigation of Transcriptional Regulatory Networks from High-Throughput Expression Data
Source: PLoS One. 2013 Jun 18;8(6):e66196. doi: 10.1371/journal.pone.0066196 (PMC3688840; doi:10.1371/journal.pone.0066196)

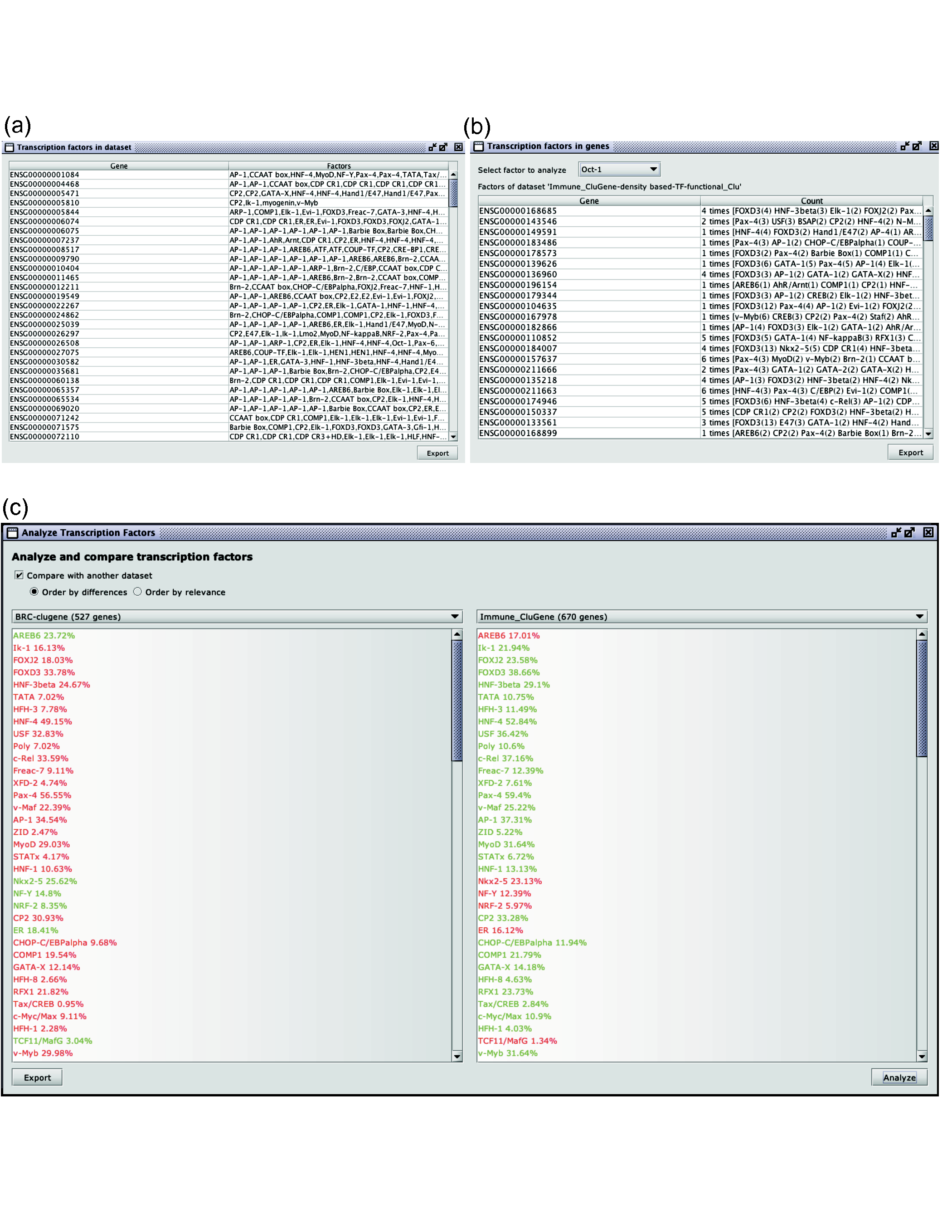

Supplement: Figure S1 — TF-based gene dataset processing. (a). For each gene present within a dataset (left panel) it is possible to identify number and types of TFBS, and consequently, what TFs can recognize them. (b). It is also possible by selecting a specific TF to search for all genes being recognized by it together with associated frequencies, a list of all TFs recognizing each gene in dataset is provided as well. (c). Distinct datasets can be compared in terms of predicted TFBs. The comparison can be ordered as a function of difference or relevance. TFs are coloured differently in the compared datasets depending on the outcome: present at an higher extent (green), present at a lower extent (red) and absent (black). (TIF) [file pone.0066196.s001.tif]

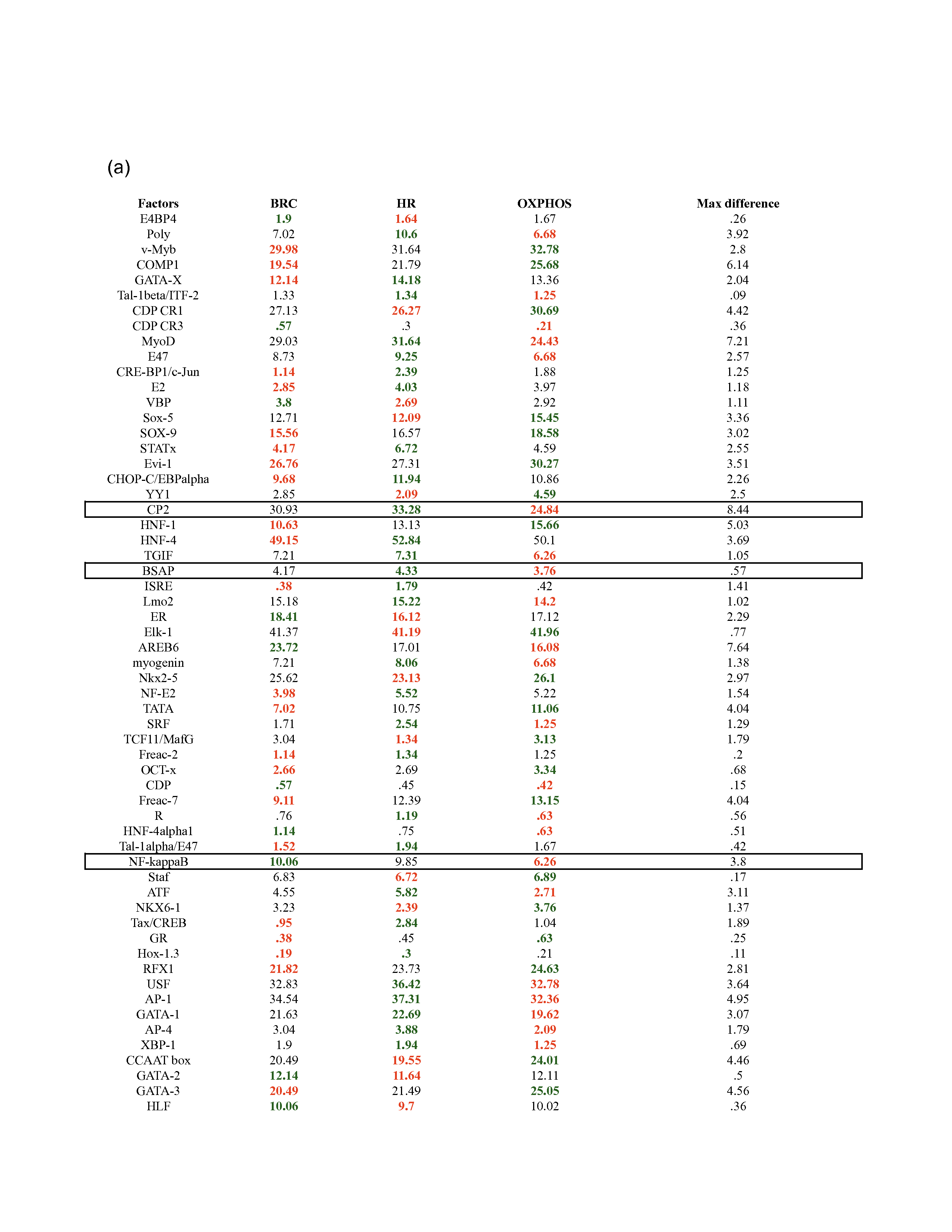

Supplement: Figure S2 — Transcription factor binding site motifs over-represented in sequences from the three DLBCL subsets (BRC, HR and OXPHOS). (a). List of TFs with associated frequencies for each DLBCL subset according to the MatchTM predictor embedded in CluGene. Annotated B cell transcription factors are boxed. TFs are coloured differently in the compared datasets depending on the outcome: Higher transcription factors binding site motifs percentages are coloured green whereas lower percentage are marked in red, absent TFBSs are coloured balck. The last column shows the maximum difference values amongst TF frequencies within the three subsets. (b). Statistical significance assessment for the results obtained with selected TFs in the OXPHOS dataset with respect to a random set of genes. Transcription factors results on the OXPHOS dataset were compared with average transcription results on 10 datasets of randomly selected genes. Ten random sets of genes were extracted form the human genome, with a number of genes same as OXPHOS. The One sample student’s t-test (SPSS) was used for the analysis. (TIF) [file pone.0066196.s002.tif]

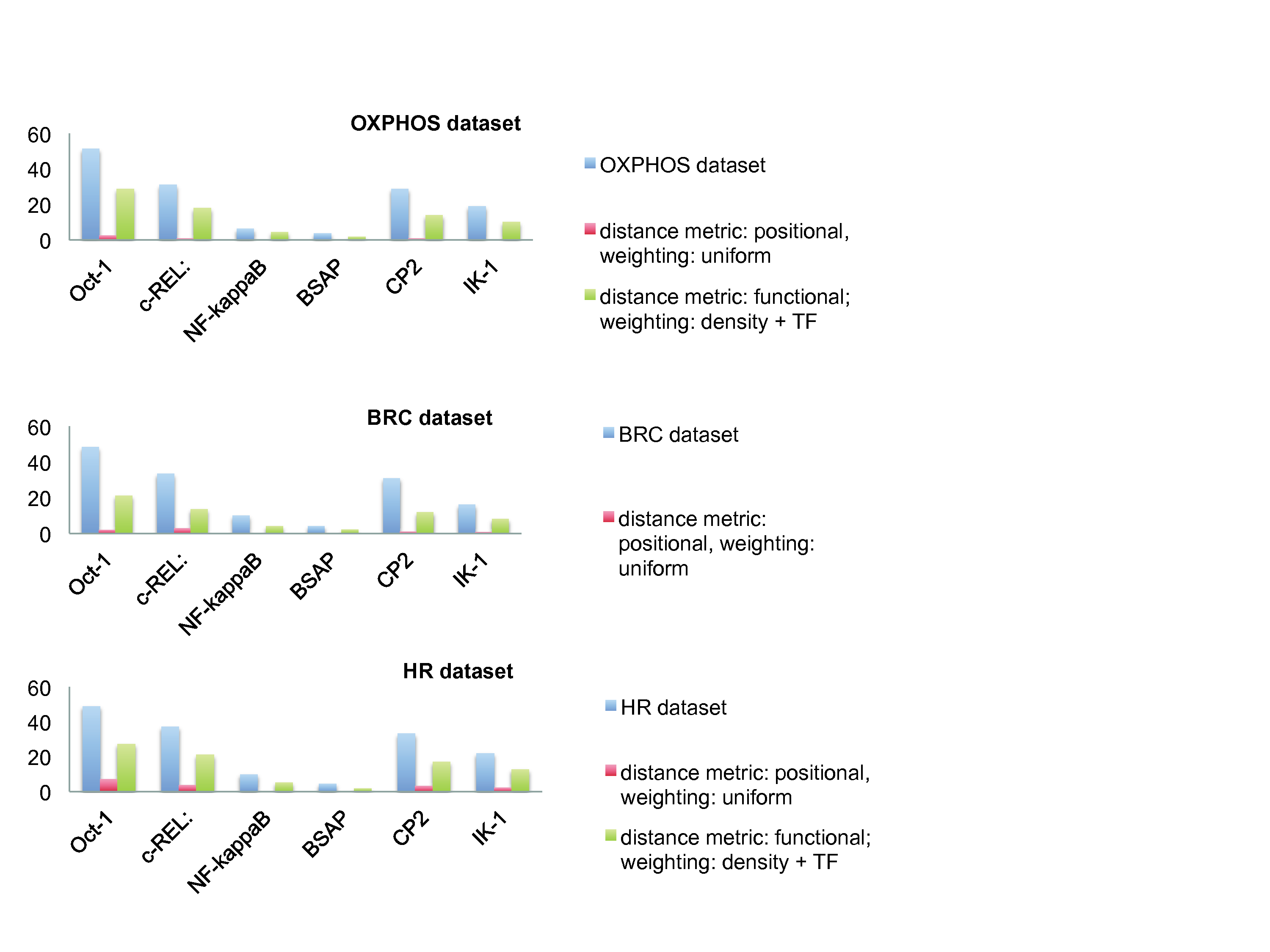

Supplement: Figure S3 — Percentages of genes that are TF targets. Number of TF target genes out of the total number of genes present within the three DLBCL subsets. A comparison between the number of TF target genes in the three DLBCL subsets and different clustering strategies output is presented. Gene clustering was performed using both the un-weighted positional clustering (Positional clustering) followed by TF prediction and the functional clustering weighted by local density and TF similarity. For the positional and functional clustering cases, the percentage refers to genes found in clusters. (TIF) [file pone.0066196.s003.tif]

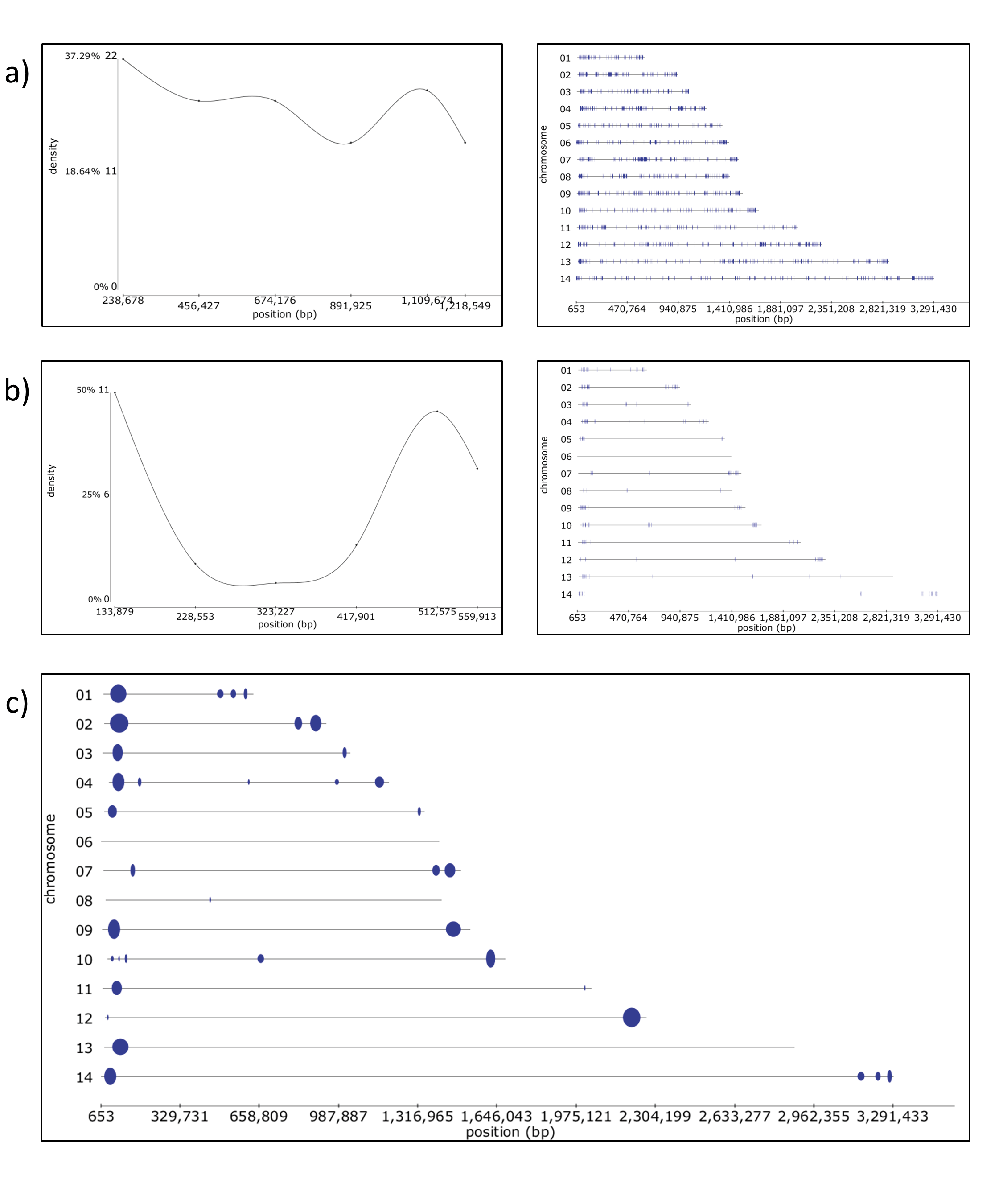

Supplement: Figure S4 — Localization of genes containing the HT motif in P. falciparum . The gene density and the cluster of genes containing specific localization attributes were predicted throughout the Plasmodium Falciparum genome using different source datasets. (a) Calculation of the gene density of genes containing the signal peptide (PlasmoDB) as localization attribute, a total of 1216 genes were considered for the analysis. The left-hand panel shows the distribution of the gene density on chromosome 5 as an example, while the right-hand panel shows the localization of genes containing the signal peptide on all chromosomes. The relative position of the individual genes (blue bars) are shown along the chromosomes (horizontal lines). The positions of the genes along the chromosomes are also indicated with a label that represents the position (in kbp). (b) Calculation of the gene density of P. falciparum genes containing the HT localization motif (PlasmoDB), a total of 214 genes were considered for the analysis, the left-hand panel shows the calculated gene density on chromosome 5 as an example, while the right-hand panel shows the localization of the genes containing the HT motif along the different chromosomes: genes tend to locate mostly at the end of the chromosomes. (c) Clustering analysis of the dataset containing the genes with the HT motif (same dataset as in (b)). The relative position and the size of individual genes clusters (blue-oval shape) are shown along the chromosomes (horizontal lines). The size of each cluster is proportional to its population size. Each cluster is also flagged with a label that indicates the position (in kbp) of the first gene. A total of 192 genes in 36 clusters were found out of the total 214. Gene clustering was performed using default positional clustering options. (TIF) [file pone.0066196.s004.tif]
