# Supplementary material for: CluGene: A Bioinformatics Framework for the Identification of Co-Localized, Co-Expressed and Co-Regulated Genes Aimed at the Investigation of Transcriptional Regulatory Networks from High-Throughput Expression Data
Source: PLoS One. 2013 Jun 18;8(6):e66196. doi: 10.1371/journal.pone.0066196 (PMC3688840; doi:10.1371/journal.pone.0066196)
Supplement: Text S1 — Supplementary information about the CluGene software. Download details; system requirements; third-party libraries used by CluGene; genome and transcription factor online databases accessible from within the software; technical details on the clustering algorithms available in CluGene. (DOCX) [file pone.0066196.s005.docx]

**Supplementary CluGene information**

CluGene is a software freely available on the website of the Bioinformatics Laboratory (BioInfoLab) at the University of Perugia (http://bioinfolab.unipg.it/). It is written in Java, and runs on any operating system (Windows, Mac or Linux) where the Java Runtime Environment (JRE) is installed (version 1.6 or higher is required). CluGene is implemented using the Java Web Start technology that allows it to be automatically downloaded and installed as a standalone application. It will run as a normal application but – if an Internet connection is available - it will also automatically check and install updates as needed.

CluGene makes use of the following free open source 3^rd^ party libraries (included in the CluGene software):

- OpenCSV (http://opencsv.sourceforge.net/): a library that allows CluGene to import and export dataset in the comma separated value format (CSV);
- the Apache Mathematics library (http://commons.apache.org/math/): a library for mathematical and statistical functions;
- HSQLDB (http://hsqldb.org/): a portable relational database management system for Java; used by CluGene to provide persistence of genome and transcription factors data for all used organisms, database of a particular organism will be downloaded only when needed the first time and will be automatically updated as needed;
- the Apache POI (http://poi.apache.org/): a library for handling Microsoft Excel documents; used for importing Excel files and for generating reports in Excel format;

Data for genomes and transcription factors have been collected by CluGene from public online services and made available in the CluGene database for easy and fast access. CluGene makes use of BioMart ver. 0.7 (<http://www.biomart.org//>) for the information about genes (e.g., positions, strands, length, etc…) and promoter sequences, while it uses different online services for the transcription factors information:

- Match™ (<http://www.gene-regulation.com/cgi-bin/pub/programs/match/bin/match.cgi>)
- Alibabà (<http://www.gene-regulation.com/pub/programs/alibaba2/index.html>)
- TFSearch (<http://www.cbrc.jp/research/db/TFSEARCH.html>)

**Clustering algorithms details**

CluGene supports configurable positional clustering algorithms with different gene distance weighting strategies. While uniform weighing does not modify the algorithm, density and expression factor based work as follow:

- *density based:* The same distance in base pair (bp) between two genes may be more relevant if found in chromosomal regions with low gene density. In terms of clustering, this implies that in densely populated areas of the chromosome, genes must be closer to each other to form meaningful clusters. To compute this type of weight, first a density function is computed by exploring each chromosome with a moving window of width w = 1.000.000 bp. The window is moved at discrete steps of side m = w/2 so that a uniform, discrete sampling of (n/m)+1 equally spaced points is obtained for a chromosome of size n. At each sampling position, the weight is defined as 1/(n.genes in the window); the final discrete function can be made continuous by interpolation (e.g. spline interpolation). See again fig. 1c for an example plot of a density function for a chromosome;
- *expression based:* Expression values are first categorized into five levels and corresponding reference values: *none* = 0.0, *low* = 0.5, *medium* = 1.0, *high* = 1.5, and *maximum* = 2.0. In case multiple expression values are available for each gene, it is possible to decide whether to use an average, the most frequent value, or the maximum value. A weighting function based on the similarity of expression levels is created to adhere to the following three rules: 1) the weight must be larger if two expression levels are similar; 2) the weight must be even larger if two similar expression levels are also high; 3) in proportion, the final weight must be more influenced by similarity of expression levels, and less by actual expression values. Such three rules are captured by the following formula: *weight* = *meanExpLvl*/(1+*diffExpInds*)^2^, where *meanExpLvl* is the arithmetic average of the two expression levels, and *diffExpInds* is the algebraic difference of the indices identifying the two expression levels (in the scale: 0,1,2,3,4). Notice that the 1+ part of the denominator is used to avoid a zero denominator in case of equal expression levels (which would lead to an infinite weight), while the exponent is used to guarantee a faster decay of the weight as the expression level difference increases. This weighting approach takes O(n*m) to compute all expression levels, where n is the number of genes and m the number of expression profiles. Usually, n is very large while m is small (0-5), so that the complexity is still O(n).

*Neighborhood model and clustering statistics.* CluGene implements an algorithm to test statistical significance of the computed clusters originally defined by Li et al [[21](#_ENREF_21)]. In a sense, this is similar to the previous comparison to a random set. For each chromosome, the mean number of adjacent genes in a random arrangement is computed as:

$$\mu=\left(M-T+1\right)T/M$$

and the standard deviation:

$$\sigma=\sqrt{\left(M-T+1\right)\left(M-T\right)T\left(T-1\right)/\left(M^{2}\left(M-1\right)\right)}$$

where M is the number of genes in the chromosome and T the number of clustered genes.
